# Clinical Experience With Thoracic Segmental Spinal Anesthesia in High‐Risk Surgical Patients

**DOI:** 10.1155/cria/7430380

**Published:** 2025-11-29

**Authors:** Alhareth M. Amro, Tasnim S. Kurdiahirbawi, Islam Frijat, Sufian M. Alrajabi, Mohammad A. Abudayyeh, Yahya M. Aljuba, Majde G. Hamamdh

**Affiliations:** ^1^ Faculty of Medicine, Al-Quds University, Jerusalem, State of Palestine, alquds.edu; ^2^ Department of Anesthesia and Critical Care, Al Ahli Hospital, Hebron, State of Palestine, ahlihospital.com

**Keywords:** case series, comorbidities, general anesthesia alternative, high-risk surgery, regional anesthesia, thoracic spinal anesthesia

## Abstract

**Background:**

General anesthesia (GA) can pose significant risks in patients with advanced comorbidities. Thoracic segmental spinal anesthesia (TSSA) is an underutilized alternative that offers hemodynamic stability and enhanced postoperative recovery. This case series highlights the application of TSSA in three high‐risk surgical patients with complex cardiovascular, renal, or oncologic conditions.

**Case Presentation:**

We describe three patients (ASA Classes III‐IV) undergoing laparoscopic or endourological procedures who received TSSA instead of GA due to high anesthetic risk. Each patient received isobaric levobupivacaine combined with dexmedetomidine via thoracic or thoracolumbar spinal injection. All patients remained awake, hemodynamically stable, and spontaneously breathing throughout their procedures. No sedation, vasopressor support, airway intervention, or ICU admission was required. Postoperative pain was minimal, with early ambulation and no reported complications.

**Conclusion:**

TSSA is a safe and effective anesthetic technique for selecting high‐risk patients undergoing abdominal or urological surgery. It provides excellent intraoperative conditions, avoids the systemic effects of GA, and facilitates rapid recovery. With proper technique and patient selection, TSSA offers a valuable alternative to GA in complex surgical cases.

## 1. Introduction

The choice of anesthetic procedure is critical in improving outcomes, especially for patients with numerous comorbidities undergoing surgical procedures [1]. While general anesthesia (GA) is widely used, it presents significant risks in the elderly and high‐risk patients, particularly those with compromised cardiovascular, renal, or pulmonary function. The consequences include increased intensive care unit (ICU) hospitalizations, postoperative cognitive dysfunction, hemodynamic instability, and longer recovery [2].

Thoracic segmental spinal anesthesia (TSSA) has developed as a specialized regional approach that has multiple benefits over GA in certain clinical settings [3]. Other advantages include better pain control than opioids and an overall decrease in opioid need before or after a procedure, which reduces the potential side effects of these drugs. Furthermore, bowel function recovers more quickly, and gastrointestinal complications, such as postoperative ileus, nausea, vomiting, and constipation, are significantly reduced, resulting in shorter hospital stays and higher patient satisfaction [3].

GA, particularly with tracheal intubation and intermittent positive pressure ventilation (IPPV), is associated with an increased risk of postoperative pulmonary complications (PPCs), especially in patients with compromised cardiopulmonary function. These include atelectasis, pneumonia, and impaired gas exchange, which can increase morbidity and length of stay. In contrast, neuraxial techniques like TSSA preserve spontaneous ventilation and diaphragmatic function, reducing the risk of PPCs. Evidence from systematic reviews and prospective multicenter studies supports a lower incidence of PPCs with regional anesthesia, showing reductions from 7.5% to 2.0% in some high‐risk populations [4].

To date, only a limited number of reports have described the use of TSSA in laparoscopic and endourological surgeries, as these procedures are traditionally performed under GA due to pneumoperitoneum and patient positioning requirements. Our series contributes to this emerging evidence by demonstrating that, with proper technique and patient selection, TSSA can be safely and effectively applied in such surgical settings. Despite its benefits, TSSA is underutilized, mainly due to safety concerns when injecting at thoracic levels. However, expanding evidence supports its feasibility and safety when performed by expert hands using appropriate technique.

Case selection criteria: Patients were considered suitable for TSSA when GA was judged to carry substantial risk due to major systemic comorbidities such as cardiovascular disease, chronic kidney disease (CKD), or active malignancy. The decision to perform TSSA was reached after a multidisciplinary discussion between the anesthesiology and surgical teams.

Exclusion criteria included patient refusal, coagulopathy or ongoing anticoagulant therapy, local infection at the puncture site, spinal deformity or prior thoracic spine surgery, known allergy to local anesthetics, and severe anxiety or inability to cooperate during an awake procedure. All patients provided written informed consent after a detailed explanation of the technique, benefits, alternatives, and potential risks.

This case series presents three high‐risk patients who successfully underwent laparoscopic and endourological procedures under TSSA, demonstrating its value as a safe, effective, and patient‐centered alternative to GA. While our previous work demonstrated the feasibility of TSSA in high‐risk patients undergoing open abdominal surgery, the current case series highlights its novel application in laparoscopic and endourological procedures surgical settings where GA is typically considered standard due to pneumoperitoneum and positioning requirements.

## 2. Case Presentation 1

We report the case of an 84‐year‐old obese male (body mass index (BMI): 30.9; weight: 89 kg; and height: 170 cm) with a history of type 2 diabetes mellitus (T2DM) complicated by retinopathy (total blindness), hypertension (HTN), stage 4 CKD with baseline creatinine 4–4.5 mg/dL, and coronary artery disease medically managed without percutaneous intervention due to high risk of contrast‐induced nephropathy and patient refusal. The patient was classified as American Society of Anesthesiologists (ASA) Physical Status IV.

The patient presented with generalized weakness, cough, and decreased oral intake for several days. He had received IV fluids at a governmental hospital and was referred to our ICU as a suspected case of sepsis. On examination, he appeared ill and dehydrated, conscious and oriented (GCS 12). Chest auscultation revealed an irregular heart rhythm and decreased air entry bilaterally. The patient’s cough was mild and nonproductive, and it was managed with preoperative nebulization using bronchodilators and normal saline. During the operation, humidified oxygen was delivered via face mask at 6 L/min to minimize airway irritation. No systemic cough suppressants or opioids were administered, as the symptoms remained controlled and did not interfere with the surgical procedure. Vital signs were stable: blood pressure (BP) 128/79 mmHg, heart rate (HR) 94 bpm, oxygen saturation (SpO_2_) 95% on room air, respiratory rate (RR) 18 breaths/min, and temperature 37.2°C. ECG confirmed atrial fibrillation.

Initial laboratory investigations revealed leukocytosis with neutrophilia, macrocytic anemia, acute kidney injury on top of CKD, and severe electrolyte disturbances, including hyperkalemia and hyponatremia. Inflammatory markers were significantly elevated, and arterial blood gases were consistent with high anion gap metabolic acidosis. The full preoperative laboratory panel is summarized in Table 1.

**Table 1:** Preoperative laboratory findings for Case 1.

Parameter	Result	Reference range	Interpretation
White blood cell count (WBC)	51 K/μL (↑)	4.0–10.0 K/uL	Leukocytosis
Hemoglobin (Hb)	8.7 g/dL (↓)	13.5–17.5 g/dL	Macrocytic anemia
Platelet count	179 K/uL	150–400 K/uL	Normal
Blood urea nitrogen (BUN)	157 mg/dL	7–20 mg/dL	Likely elevated (CKD, sepsis)
Creatinine	5.57 mg/dL	0.7–1.3 mg/dL	Stage 4 CKD
Sodium	130 mEq/L (↓)	135–145 mEq/L	Hyponatremia
Potassium	8 mEq/L (↑)	3.5–5.0 mEq/L	Hyperkalemia
pH	7.23	7.35–7.45	Severe acidosis
CRP	316 mg/L	Up to 6 mg/L	Consistent with sepsis
HbA1c	10% (↑)	< 7% (T2DM target)	Uncontrolled
Blood glucose (random)	180–300 mg/dL	< 140 mg/dL (nonfasting)	Hyperglycemia

Chest CT revealed bilateral atypical pneumonia, and abdominal ultrasound demonstrated a distended gallbladder measuring 8.5 × 3.5 cm with stones impacted at the neck. Echocardiography showed preserved systolic function (ejection fraction [EF] 60%) and mild aortic, tricuspid, and mitral regurgitations. A diagnosis of sepsis secondary to pneumonia and acute cholecystitis was made. Following intensive supportive care, including fluid correction, electrolyte stabilization, and antibiotic therapy, the patient’s condition gradually improved. After several days, repeat investigations confirmed clinical and laboratory improvement, and the patient was cleared for laparoscopic cholecystectomy.

Given the patient’s stage 4 CKD, sepsis, and limited physiological reserve, GA was considered high risk due to potential hemodynamic instability and nephrotoxic medication exposure. TSSA was chosen to avoid mechanical ventilation, preserve spontaneous breathing, and minimize systemic drug burden.

In the operating room, standard ASA monitoring was initiated. Initial vitals were stable: BP 142/90 mmHg, HR 95 bpm, SpO_2_ 95%. In the sitting position, the T8‐T9 interspace was accessed using the median approach with a 25‐gauge pinpoint spinal needle under sterile technique. After confirmation of CSF flow, the subarachnoid block was administered using 1.5 mL of 0.5% isobaric levobupivacaine (7.5 mg), along with 5 mcg of dexmedetomidine. The patient was then placed supine. The sensory block level achieved was T4–T6 up and L2‐L3 down, confirmed by pinprick and cold sensation testing within 5–10 min of injection.

No sedation was required intraoperatively. The patient remained fully awake, hemodynamically stable, and cooperative throughout the procedure. Pneumoperitoneum was created with CO_2_ after Veress needle insertion, and a standard four‐port laparoscopic cholecystectomy was performed. The critical view of safety was achieved, and the gallbladder was dissected retrogradely from the liver bed. The procedure lasted 45 min, with no intraoperative complications.

Intraoperative fluid management included a total of 500‐mL crystalloid infusion, with no need for blood transfusion or vasopressors. The patient breathed spontaneously with 6 L/min oxygen via a face mask. Vital signs remained stable: systolic blood pressure (SBP) ranged between 110 and 142 mmHg, and diastolic blood pressure (DBP) ranged between 63 and 90 mmHg. HR is 70–110 bpm, and SpO_2_ improved to 99%.

Motor and sensory block persisted for approximately 2 h, after which the patient gradually regained full lower limb function. Postoperative pain was minimal (VAS score < 2/10), and no nausea, hypotension, urinary retention, or neurological symptoms were observed. The patient ambulated the same evening and was monitored in the surgical ward without the need for ICU care. At discharge, the patient was alert, mobile, and satisfied with his anesthetic and surgical experience. This case demonstrates that TSSA can be a safe and effective anesthetic alternative in elderly, high‐risk patients undergoing laparoscopic cholecystectomy.

## 3. Case Presentation 2

A 57‐year‐old female, chronic smoker with a BMI of 34.4 (weight: 85 kg and height: 157 cm), presented to our hospital with abdominal distention and right flank pain. She had a history of T2DM, paroxysmal atrial fibrillation, and CKD with a baseline creatinine of 1.8 mg/dL. Three months prior to admission, she was diagnosed with metastatic ovarian cancer after presenting with progressive abdominal distention and daily discomfort. A CT scan at that time revealed a 9.5 × 9.2 cm right adnexal mass, moderate ascites, multiple pathologically enlarged intra‐abdominal lymph nodes, two uterine fibroids, and three hepatic hemangiomas. A core biopsy confirmed the diagnosis of ovarian adenocarcinoma, and she began chemotherapy, with her first cycle administered 10 days prior to this admission. Her ASA physical status was classified as class IV.

She was not on any medications other than metformin and had no known drug allergies. On physical examination, she appeared ill and pale, though conscious and oriented. Her abdomen was tense and distended with shifting dullness, and she exhibited +2 bilateral lower limb edema. Cardiopulmonary examination revealed no abnormalities. Vital signs were stable: BP 113/67 mmHg, HR 86 bpm, SpO_2_ 95% on room air, RR 16 breaths per minute, and temperature 38.0°C. Laboratory investigations on admission revealed leukopenia (WBC 0.78 K/μL), anemia (Hb 8.9 g/dL), platelet count of 228 K/μL, markedly elevated BUN (106 mg/dL), and a creatinine level of 4.5 mg/dL—significantly above her baseline. Hemoglobin A1c was 6.8%. ECG showed a regular rhythm with no acute changes, and chest radiography was unremarkable (Table 2).

**Table 2:** Preoperative laboratory findings for Case 2.

Parameter	Result	Reference range	Interpretation
White blood cell count (WBC)	0.78 K/μL (↑)	4.0–10.0 K/uL	Severe leukopenia (postchemo)
Hemoglobin (Hb)	8.9 g/dL (↓)	13.5–17.5 g/dL	Anemia
Platelet count	228 K/uL	150–400 K/uL	Normal
Blood urea nitrogen (BUN)	106 mg/dL (↑)	7–20 mg/dL	Severe renal impairment
Creatinine	4.5 mg/dL	0.7–1.3 mg/dL	Acute on CKD
Sodium	140 mEq/L (↓)	135–145 mEq/L	Normal
Potassium	4.4 mEq/L (↑)	3.5–5.0 mEq/L	Normal
pH	Not specified	7.35–7.45	_____
CRP	Not specified	—	_____
HbA1c	6.8%	< 7% (T2DM target)	Controlled
Blood glucose (random)	121 mg/dL	< 140 mg/dL (nonfasting)	Normal

*Note:* Not specified = laboratory test not done routinely and only at special request.

A contrast‐enhanced abdominal CT scan was performed and showed moderate ascites and severe right‐sided hydronephrosis, for which urological intervention was indicated. A right‐sided double *J* stent insertion was scheduled for the following day. Although the planned endourological procedure was short, the patient had acute‐on‐chronic renal impairment and chemotherapy‐induced leukopenia. These factors made GA less desirable, and the need for higher dermatomal coverage than what lumbar spinal could reliably provide led us to prefer TSSA.

**Figure 1:**
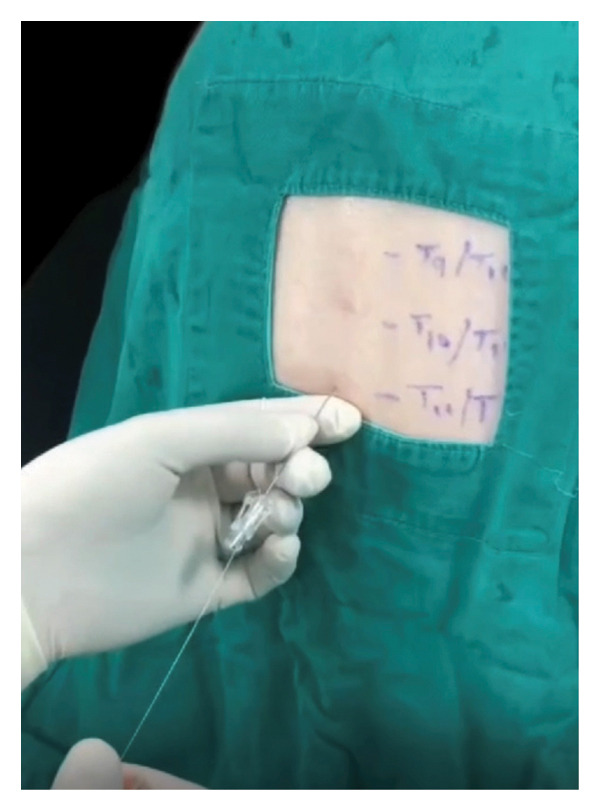
Midline needle insertion technique at the T8‐T9 interspace using a 25‐gauge spinal needle for thoracic segmental spinal anesthesia. The patient is positioned sitting, and a sterile field is maintained.

In the operating room, full ASA monitoring was applied. Preanesthesia vitals remained stable: BP 105/60 mmHg, HR 86 bpm, and SpO_2_ 95%. With the patient in the sitting position, the T11‐T12 interspace was identified and disinfected thoroughly. Local infiltration with 2% lidocaine was administered. Using the median approach, a 25‐gauge spinal needle was inserted, and cerebrospinal fluid (CSF) was obtained on the first attempt. Intrathecal injection of 1.5 mL of 0.5% isobaric levobupivacaine (7.5 mg) combined with 5‐mcg dexmedetomidine was performed slowly over 10 s and without complications (Figure 1).

Sensory block to the T8–L4 dermatomes was achieved within 5 min, assessed by cold sensation and pinprick testing. No sedation was needed, and the patient reported comfort throughout the procedure. Motor block was mild and appropriate for the surgical area. The patient was placed in the lithotomy position, and cystoscopy was performed. A 6 French double *J* stent was inserted into the right ureter under fluoroscopic guidance without difficulty. The surgery lasted approximately 45 min, during which the patient remained fully awake, breathing spontaneously with stable vitals, and without the need for supplemental oxygen or vasoactive medications. Estimated blood loss was negligible, and 500 mL of crystalloid was administered intravenously.

Postoperatively, the patient was transferred to the surgical department for observation. She regained full motor function within 2 h, experienced minimal pain (VAS score 1‐2), and was ambulated on the same day. No nausea, hypotension, headache, or neurologic complications were observed. She remained hemodynamically stable and satisfied with the anesthetic experience.

This case highlights the effective use of TSSA in a high‐risk, immunocompromised patient undergoing endourological intervention. Regional anesthesia techniques such as this offer a safer alternative to GA in select populations, particularly in the setting of renal impairment and active oncologic treatment.

## 4. Case Presentation 3

A 67‐year‐old male patient with a BMI of 36.3 (weight: 105 kg and height: 170 cm) presented with a two‐month history of intermittent, colicky right upper quadrant abdominal pain exacerbated by fatty meals and alleviated by fasting and analgesics. His medical history included T2DM, HTN, ischemic heart disease (IHD) with three percutaneous coronary interventions (PCIs), the most recent in 2019, coronary artery bypass grafting (CABG) in 1996, benign prostatic hyperplasia (BPH), and L4‐L5 discectomy in 2022. His medication regimen comprised metformin 850 mg, bisoprolol 5 mg, atorvastatin 40 mg, aspirin 100 mg, tamsulosin 0.4 mg, furosemide 40 mg, and dapagliflozin 10 mg. He reported an allergy to sulfa drugs.

On examination, the patient appeared ill and pale but was conscious and oriented. His abdomen was tense and distended, with bilateral +2 pitting edema in the lower limbs. Cardiopulmonary examination revealed no additional sounds or murmurs. Vital signs were: BP 110/60 mmHg, HR 65 bpm, RR 14 breaths per minute, SpO_2_ 97% on room air, and temperature 38.0°C.

Laboratory investigations revealed a white blood cell (WBC) count of 8.4 K/μL, hemoglobin level of 17.05 g/dL, hematocrit at 52.5%, and platelet count of 156.1 K/μL. Renal function tests showed a blood urea nitrogen (BUN) of 22 mg/dL and creatinine at 0.9 mg/dL. Glycemic assessment indicated an HbA1c of 5.4% and a random blood sugar of 121 mg/dL. Electrolyte analysis revealed sodium at 140 mEq/L and potassium at 4.4 mEq/L. These findings suggest stable renal function and adequate glycemic control (Table 3).

**Table 3:** Preoperative laboratory findings for Case 3.

Parameter	Result	Reference range	Interpretation
White blood cell count (WBC)	8.4 K/uL	4.0–10.0 K/uL	Normal
Hemoglobin (Hb)	17.05 g/dL (↑)	13.5–17.5 g/dL	Elevated
Platelet count	156.1 K/uL	150–400 K/uL	Normal
Blood urea nitrogen (BUN)	22 mg/dL (↑)	7–20 mg/dL	Mildly elevated
Creatinine	0.9 mg/dL	0.7–1.3 mg/dL	Normal
Sodium	140 mEq/L	135–145 mEq/L	Normal
Potassium	4.4 mEq/L	3.5–5.0 mEq/L	Normal
pH	Not specified	7.35–7.45	—
CRP	Not specified	—	—
HbA1c	5.4%	< 7% (T2DM target)	Excellent control
Blood glucose (random)	121 mg/dL	< 140 mg/dL (nonfasting)	Normal

*Note:* Not specified = laboratory test not done routinely and only at special request.

Electrocardiography (ECG) demonstrated a right bundle branch block (RSR pattern) with *Q* waves in anteroseptal leads. Chest x‐ray was unremarkable. Abdominal ultrasound revealed multiple gallbladder stones without signs of cholecystitis and an enlarged prostate. Transthoracic echocardiography indicated an EF of 40%, with septal, anterior, and apical hypokinesia, mild mitral and tricuspid regurgitation, and a pulmonary artery pressure (PAP) of 27 mmHg at rest.

He was classified as ASA Physical Status Class III. This patient’s history of IHD, reduced EF (35%–40%), and prior coronary interventions placed him at elevated risk for hypotension and myocardial depression under GA. TSSA allowed a controlled, segmental block with minimal impact on preload and systemic circulation.

In the operating room, standard monitoring was applied, revealing BP 105/57 mmHg, HR 67 bpm, and SpO_2_ 96%. With the patient in the sitting position, the T8‐T9 interspace was identified and prepared under sterile conditions. Local infiltration with 2% lidocaine was administered. Using the paramedian approach, a 25‐gauge spinal needle was inserted, and clear CSF was obtained on the first attempt. Intrathecal injection of 1.5 mL of 0.5% isobaric levobupivacaine (7.5 mg) combined with 5‐mcg dexmedetomidine was performed slowly over 10 s.

A sensory block from T6 to L3 was achieved within 5 min, confirmed by cold sensation and pinprick testing. No significant hemodynamic changes occurred postanesthesia. The patient was positioned with a head‐up tilt toward the surgeon’s side. Pneumoperitoneum was established, and laparoscopic cholecystectomy was completed uneventfully over 40 min. The patient remained awake, breathing spontaneously, and hemodynamically stable throughout the procedure without complications.

Postoperatively, the patient was transferred to the surgical department for observation. He reported minimal pain (Visual Analog Scale score 1‐2) and regained full motor function within 2 h. No postoperative nausea, vomiting, or neurologic complications were observed. He was mobilized on the same day and discharged in stable condition after an uneventful recovery.

This case illustrates the successful use of TSSA in a high‐risk patient undergoing laparoscopic cholecystectomy. TSSA offers advantages such as stable hemodynamics, effective analgesia, and reduced postoperative complications compared with GA, particularly in patients with significant cardiopulmonary comorbidities. TSSA is a viable and effective anesthetic technique for laparoscopic cholecystectomy in patients with multiple comorbidities, providing stable intraoperative conditions and favorable postoperative outcomes.

The three patients in this series were classified as ASA Physical Status Classes III and IV and presented with significant comorbidities, including cardiovascular disease, CKD, and/or active malignancy. In each case, TSSA was selected over GA to minimize systemic impact, reduce perioperative risk, and promote rapid postoperative recovery.

Despite differences in surgical indication and individual comorbidity profiles, the anesthetic approach was similarly structured: a thoracic or thoracolumbar spinal block using isobaric levobupivacaine with intrathecal adjuvant. All patients remained awake, spontaneously breathing, and hemodynamically stable throughout the procedures. None required sedation, vasopressors, airway management, or ICU‐level care postoperatively. Pain control was excellent, and all patients ambulated on the same day of surgery. A comparative summary of the anesthetic details and outcomes is provided in Table 4.

**Table 4:** Comparison of anesthetic techniques and outcomes in all three cases.

Case no.	Age/Gender	ASA class	Surgery type	Level of LA injection	LA and adjuvants injected	Sensory block level	Duration of sensory/motor block	Surgery duration (min)	Sedation used	Airway support	Hemodynamic stability	Postop recovery	Time to ambulation for each patient.
1	84/M	IV	Laparoscopic cholecystectomy	T8‐T9	7.5‐mg isobaric levobupivacaine + 5‐mcg dexmedetomidine	T4–T10	Sensory: 2 h Motor: 100 min	45	No	Spontaneous (O_2_ mask)	Stable	Mobilized same day	1 Day
2	57/F	IV	Ureteric stent insertion (endourological)	T11‐T12	7.5‐mg isobaric levobupivacaine + 5‐mcg dexmedetomidine	T8–S2	Sensory: 1.8 h Motor: 95 min	45	No	Room air	Stable	Mobilized same day	1 Day
3	67/M	III	Laparoscopic cholecystectomy	T8‐T9	7.5‐mg isobaric levobupivacaine + 5‐mcg dexmedetomidine	T6–T12	Sensory: 2.1 h Motor: 105 min	40	No	Room air	Stable	Mobilized same day	1 Day

## 5. Discussion

TSSA has emerged as a valuable alternative to GA in selecting high‐risk surgical patients [5]. This case series demonstrates the successful use of TSSA in three individuals with significant comorbidities, including CKD, IHD, diabetes, and malignancy. In each case, TSSA was chosen to mitigate the systemic impact of GA, reduce perioperative risk, and facilitate faster postoperative recovery. The outcomes were consistently favorable: all patients remained awake, spontaneously breathing, and hemodynamically stable during surgery, with no need for sedation, vasopressor support, or airway intervention.

Beyond hemodynamic benefits, a major advantage of TSSA is the reduction in pulmonary complications compared with GA. Mechanical ventilation under GA disrupts diaphragmatic mechanics, reduces functional residual capacity (FRC), and alters ventilation–perfusion matching, especially in elderly or high‐risk patients. Even with protective ventilation strategies, GA remains a risk factor for PPCs. Neuraxial anesthesia allows patients to breathe spontaneously, enhancing ventilation of dependent lung regions and preserving pulmonary mechanics. This physiological advantage has been linked to reduced rates of PPCs in large studies, supporting the use of TSSA in appropriate surgical candidates [4].

The hemodynamic stability observed in our patients, with no requirement for supplemental vasopressors, is explained by the physiological characteristics of TSSA. Specifically, the restricted segmental blockade achieved by thoracic spinal anesthesia minimizes sympathetic blockade compared with traditional lumbar spinal anesthesia. This results in less vasodilation and reduced preload compromise, maintaining cardiovascular stability and avoiding significant drops in blood pressure [6].

There was coordination with the surgical team to ensure that the insufflation pressure within the abdomen did not exceed 10–12 mmHg, minimizing diaphragmatic splinting and patient discomfort during pneumoperitoneum. An interesting and unexpected observation in our two laparoscopic cases was the complete absence of referred shoulder pain, which is commonly attributed to diaphragmatic irritation transmitted via the phrenic nerve (C3–C5). Our thoracic spinal block did not extend to cervical dermatomes, making this finding noteworthy. We hypothesize that several factors may have contributed to the lack of shoulder pain, including the maintenance of low pneumoperitoneum pressures (≤ 12 mmHg), limited peritoneal traction, and possible central analgesic effects of intrathecal dexmedetomidine. Although alpha‐2 agonists are known to modulate visceral pain pathways, the evidence for their role in preventing referred diaphragmatic pain is limited, and our sample size does not allow for firm conclusions. We acknowledge this limitation and suggest that further studies are needed to explore this observation [7, 8].

Notably, sedation was deliberately withheld in all three cases. Given the patients’ advanced comorbidities particularly cardiac and renal dysfunction our primary objective was to minimize the risk of respiratory depression, hemodynamic instability, and delayed recovery. Although the intrathecal dexmedetomidine dose used (5 mcg) was relatively low, we acknowledge that its central alpha‐2 adrenergic effects may have contributed to a mild sedative or anxiolytic state, which could have supported patient comfort during surgery. All patients remained awake, communicative, and cooperative throughout the procedures, without signs of discomfort or the need for conversion to GA. This experience suggests that a sedation‐free TSSA approach is feasible in well‐prepared, high‐risk individuals. Nevertheless, we recognize that light sedation may still be beneficial in selected patients, particularly those with anxiety or limited tolerance for awareness during surgery.

In TSSA, the anesthetic drug used is critical to provide a controlled, segmental sensory block while maintaining hemodynamic stability and lesser vasopressor use and early ambulation [9]. Isobaric levobupivacaine is commonly preferred for TSSA due to its predictable spread, slower onset, and prolonged duration of action compared with other local anesthetics [10]. Unlike hyperbaric solutions, isobaric formulations tend to remain localized around the site of injection, allowing for more precise control of dermatomal spread, which is essential for thoracic applications where excessive cephalad or caudal distribution could result in high spinal block or unnecessary motor block [11]. Adjuvants such as dexmedetomidine, fentanyl, or clonidine are often added intrathecally to enhance the quality and duration of the block, reduce the required dose of local anesthetic, and provide superior postoperative analgesia without increasing systemic side effects [12].

In our case series, a consistent anesthetic regimen was employed across all three patients: 1.5 mL of 0.5% isobaric levobupivacaine (7.5 mg) combined with 5 mcg of dexmedetomidine, administered at either the T8‐T9 or T11‐T12 interspaces. This combination facilitated rapid onset of an adequate sensory block covering the necessary dermatomes for laparoscopic and endourological procedures, while preserving motor function in the lower limbs. The use of dexmedetomidine as an intrathecal adjuvant significantly enhanced intraoperative analgesia, prolonged the duration of sensory blockade, and contributed to stable intraoperative hemodynamics in our patients. Dexmedetomidine has been increasingly favored as an additive to spinal anesthesia due to its synergistic analgesic effects when combined with local anesthetics. Several studies have demonstrated these benefits, including enhanced sensory and motor block characteristics, prolonged postoperative analgesia, and reduced analgesic requirements postoperatively [13, 14]. These effects likely result from dexmedetomidine’s alpha‐2 adrenergic receptor agonism, which potentiates local anesthetic action by inhibiting nociceptive transmission at spinal cord levels. Importantly, none of the patients required supplemental opioids, sedation, or vasopressor support, and all reported minimal postoperative pain, supporting the efficacy and safety of this anesthetic approach in high‐risk surgical populations.

Although the patients in this series presented with stable vital signs preoperatively, each had significant underlying comorbidities including advanced CKD, IHD, or chemotherapy‐induced immunosuppression that elevated their risk for perioperative complications under GA. TSSA was selected to avoid mechanical ventilation, reduce systemic anesthetic exposure, and provide precise dermatomal coverage. In contrast to lumbar spinal anesthesia, TSSA offers better control for midabdominal and pelvic procedures while minimizing cephalad spread and hemodynamic instability. These considerations guided our anesthetic choice in each case and support the value of TSSA in carefully selected high‐risk patients.

TSSA remains underutilized in clinical practice because of concerns about spinal cord injury when injected at thoracic levels. However, growing evidence indicates it is safe in the hands of professionals [15]. Previous studies, such as those by Elakany et al. and Aljuba et al., found equally positive outcomes in patients undergoing breast or abdominal procedures, particularly in high‐risk or elderly populations [3]. The present series reinforces these findings by extending the application of TSSA to laparoscopic and endourological procedures in patients with ASA Class III status. The hemodynamic stability and minimal postoperative morbidity observed in our cases highlight the physiologic advantages of avoiding GA in patients with compromised cardiac or renal function.

Each case presented unique challenges that illustrate the versatility of TSSA. In the first case, an elderly male with sepsis and stage 4 CKD successfully underwent laparoscopic cholecystectomy without deterioration in renal function or cardiovascular status. The second case involved a patient with advanced ovarian cancer, chemotherapy‐induced leukopenia, and acute on chronic renal failure. TSSA offered a low‐risk anesthetic plan that avoided further nephrotoxicity. In the third case, a cardiac patient with reduced EF and previous coronary interventions underwent surgery safely without myocardial stress, arrhythmia, or hypotensive episodes. These examples demonstrate how TSSA can be adapted to reduce risks in complex clinical situations when GA is potentially harmful.

This case series demonstrates that TSSA, when performed by experienced clinicians with careful patient selection, offers a safe and effective alternative to GA in high‐risk surgical patients. It enables stable intraoperative conditions, rapid postoperative recovery, and reduced systemic complications. As awareness and technical proficiency grow among anesthesiologists, TSSA may assume a more prominent role in perioperative care for vulnerable patient populations. The expansion of TSSA to laparoscopic and endourological procedures raises important questions regarding technique reproducibility, safety, and training. Unlike conventional lumbar spinal anesthesia, thoracic spinal blocks require precise needle placement and segmental targeting. Future studies should explore the learning curve, standardization of technique, and structured training strategies necessary for widespread and safe clinical adoption of TSSA across diverse surgical practices.

This case series demonstrates that TSSA, when performed by experienced clinicians with careful patient selection, offers a safe and effective alternative to GA in high‐risk surgical patients. It enables stable intraoperative conditions, rapid postoperative recovery, and reduced systemic complications.

Importantly, our report adds to the limited existing literature describing the successful use of TSSA in laparoscopic and endourological surgeries procedures that are conventionally conducted under GA. These findings highlight the growing feasibility of TSSA as a viable anesthetic option beyond open surgical procedures and emphasize its potential expansion into minimally invasive surgical practice.

## 6. Conclusion

This case series highlights the feasibility, safety, and effectiveness of TSSA in high‐risk surgical patients. In all three cases, TSSA provided excellent intraoperative conditions, stable hemodynamics, and rapid postoperative recovery without the need for sedation, airway support, or ICU admission. The absence of complications and early mobilization underscore its potential as a viable alternative to GA, particularly in patients with significant cardiovascular, renal, or oncologic comorbidities. With growing clinical experience and careful patient selection, TSSA may play an increasingly important role in the anesthetic management of complex surgical patients.

## Ethics Statement

All procedures performed in this study involving human participants were conducted in accordance with the ethical standards of the institutional and national research committees and with the 1964 Helsinki Declaration and its later amendments. Ethical approval for this case series was obtained from the Institutional Review Board (IRB) at Al‐Quds University (Approval Code: 583/REC/2025). Written informed consent was obtained from all patients for participation, data collection, and publication of anonymized clinical details and accompanying images. A copy of the signed consent forms is available for review by the Editor‐in‐Chief upon request.

## Consent

Written informed consent was obtained from all patients for the publication of this case series and any accompanying images. The patients were informed that their anonymity would be preserved and that no identifying information would be published.

## Disclosure

A preprint has previously been published [16]. All authors read and approved the final version of the manuscript.

## Conflicts of Interest

The authors declare no conflicts of interest.

## Author Contributions

Alhareth M. Amro and Tasnim S. Kurdiahirbawi contributed equally to the conception, data collection, clinical analysis, literature review, and writing of the manuscript. Islam Frijat, Mohammad A. Abudayyeh, and Yahya M. Aljuba performed the anesthesia procedures and assisted in clinical case documentation. Majde G. Hamamdh supervised the clinical work and provided critical revisions to the manuscript.

## Funding

The authors received no specific funding for this work.

## Data Availability

The data supporting the findings of this study are included within the article. Additional patient information and clinical details are available from the corresponding author upon reasonable request, subject to institutional and ethical guidelines to protect patient confidentiality.
